# Highly Sensitive Localized Surface Plasmon Polariton Based D-Type Twin-Hole Photonic Crystal Fiber Microbiosensor: Enhanced Scheme for SERS Reinforcement

**DOI:** 10.3390/s20185248

**Published:** 2020-09-14

**Authors:** Manthangal Sivanesan Aruna Gandhi, Krishnamoorthy Senthilnathan, Padmanabhan Ramesh Babu, Qian Li

**Affiliations:** 1 School of Electronic and Computer Engineering, Peking University, Shenzhen 518055, China; aruna@pkusz.edu.cn; 2 School of Advanced Sciences, Vellore Institute of Technology, Vellore 632014, India; senthilnathan.k@vit.ac.in (K.S.); prameshbabu@vit.ac.in (P.R.B.)

**Keywords:** microbiosensor, stimulated emission of Raman scattering, surface plasmon resonance, localized surface plasmon resonance, sensitivity, linearity

## Abstract

The emerging development of sensing technology initiates innovative sensors achieving low-cost to facilitate practical realization. An interesting crush of the work is to propose a simple structural sensor and to analyze the different schemes of the metal coating by stimulated emission of Raman scattering (SERS) intensification. For the first time, we propose a simple geometrical photonic crystal fiber refractive index based sensor (PCF-RIBS) with three different Schemes A, B, and C, i.e., gold (A) layer-coated surface plasmon resonance (SPR) based D-type PCF-RIBS; Au with titanium-di-oxide (TiO${}_{2}$) layer-coated SPR D-type PCF-RIBS; and Au + TiO${}_{2}$ grating-coated localized surface plasmon resonance (LSPR) D-type PCF-RIBS. Characterizing the three different Schemes A, B, and C using finite element method simulation shows, a maximum wavelength sensitivity of 48,000 nm/RIU, 52,000 nm/RIU and 75,000 nm/RIU, respectively, for a wide range of analyte-refractive index from 1.33 to 1.45 and operates in the wavelength range from 500–2000 nm. Of all the Schemes, Scheme C is found to excite a relatively larger number of surface-plasmons. Eventually, it exhibits improved sensing performances compared to SPR based Schemes A and B. Consequently, it would turn out to be an appropriate candidate to detect a broad range of biological and chemical sample detection.

## 1. Introduction

The localized surface plasmon resonance (LSPR) phenomenon describes the propagation of the surface plasmons excited in nanoparticles or nanogratings whose size is smaller than that of the wavelength of the incident light. The strong photosensitive confinement and evanescent field amplification of LSPR are principally investigated for SERS wherein the enhanced electromagnetic fields around the metal nanostructure endorse a substantial Raman response of the biomolecular sample kept in contact with the metal [1]. PCFs are advantageous with respect to the structural engineering, dimensions, and tolerance to the electromagnetic field interference. With LSPR impressed on a PCF turns out to be a sensor adequate for the point-of-care sensing applications. To analyze the biological samples and SERS reinforcement, SPR and LSPR sensors are reported [2,3,4,5,6,7,8,9]. The SPR sensor based on the traditional Kretschmann configuration by attenuated total reflection investigation has achieved a sensitivity of 2459.3 nm/RIU for the $\left(n_{a}\right)$ range from 1.33–1.36 [2]. Recently, the biological and chemical sensors based on SPR and LSPR with internal and external sensing of $n_{a}$ by different sensor configurations, namely, prism, grating, and fiber-based sensors have been investigated [1,2,3,4,5]. Of all these sensor configurations, the advantages of fiber-based sensors are miniaturization, a high degree of integration and design flexibility. In particular, by engineering the geometric structure, one can tailor the effective refractive index of the core-guided mode and the surface plasmonic mode [3,4,5]. When the refractive index of the core-guided mode matches with that of the surface plasmonic mode, the resonance occurs. As a result, a part of energy from core-guided mode gets transferred to the surface plasmonic mode. The change in $n_{a}$ affects the appropriate modes, and it eventually leads to the different loss spectra of the core-guided mode. This phenomenon has been utilized to detect the analytes in the chemical and biological fields. Nevertheless, two consequential challenges obstruct the evolution of these optical fiber-based sensors.

The first significant difficulty is the manufacturing hassle about the sensor. The chemical vapor deposition (CVD) technique introduces the metallizing operation in the holes and also requires technique [10,11,12]. However, in these fiber geometries, the analyte is infiltrated and also suffers refilling issues. Due to its time-consumption and difficulty of real-time sensing, it is worthwhile to adopt the external sensing scheme [10,11,12,13]. Amongst the choices, the D-shaped fiber optic sensors are efficient in sensing the analyte under investigation (AUI) with the metal directly deposited on their exposed section [4,14,15]. The second difficulty is high refractive index-contrast measurements. Furthermore, the silica fiber-based sensors have the upper detection limit of $n_{a}$ less than 1.42 [13,14]. When $n_{a}$ is higher than the fiber-material’s refractive index, the conventional total internal reflection is achieved. We note that these fiber-based sensor geometries wherein the metallizing and filling of the fiber holes are necessary experience an exertion in real-time sensing. Recently, studies using SPR technology by internal or external sensing schemes with D-shaped structure were reported [4,15,16,17]. Thus, the external sensing scheme overcomes the internal sensing with filling the fiber-holes with various analyte via syringe (i.e., withdrawal and pumping mode). However, in external sensing, the analyte is in-contact with fiber surface. The third difficulty is thick cladding layers from these fiber sensors limit the fields to interact straightly through evanescent waves arising from core/cladding boundaries. Hence, it results in a loss of mechanical strength. In order to solve these problems in SPR based photonic crystal fiber (PCF) sensors, LSPR-based sensors are established to enhance the sensing performance along with effective size and cost [5,18,19,20]. Recent research using nanospheres achieves a maximum wavelength sensitivity of 27,000 nm/RIU [19]. In addition, by using gold grating, a maximum wavelength sensitivity of 3340 nm/RIU with the high confinement loss has been reported [4]. Furthermore, an LSPR biosensor with an improved technique for SERS response intensification has been able to provide a record wavelength sensitivity of 111,000 nm/RIU with the analyte upper detection limit of 1.43 [20]. However, the above sensor has several air-holes with different sizes in the cladding region, and hence it is difficult to fabricate.

In this paper, we intend to design a sensor that can enhance the absorption, resolution, and limit of detection (LOD) with a low confinement loss. Here, the proposed sensor works based on an external sensing scheme. The organization of the paper is as follows. In Section 2, we propose a simple geometrical D-type twin-hole PCF sensor with external sensing, which is basically a real-time sensor. We propose the sensors based on three possible schemes, namely, ‘Scheme A’, ‘Scheme B’, and ‘Scheme C’. Here, ‘Scheme A’ describes the Au coated on the D-surface, ‘Scheme B’ defines the Au with titanium-di-oxide (TiO${}_{2}$) coated on the D-surface, and ‘Scheme C’ denotes the Au + TiO${}_{2}$-grating coated on the D-surface of the sensor. The investigation of modes and physical mechanism of the proposed sensors are discussed in Section 3. For the ease of fabrication, we optimize the sensor by varying the geometrical parameters. Furthermore, we optimize the proposed sensor for operation in visible to near-IR wavelength i.e., from 500 to 2500 nm for the AUI of 1.33 to 1.45 to improve the LOD. In Section 4, we delineate the influence of metal and optimization in the proposed sensors. Section 5 discusses the fabrication tolerance of the proposed schemes. Furthermore, the sensing performances and linearity of the sensor are explained in Section 6. Finally, performance of the sensor is summarized in Section 7.

## 2. Sensor Design and Principles

### 2.1. D-Type Twin-Hole PCF-RIBS Microbiosensor

**Figure 1:**
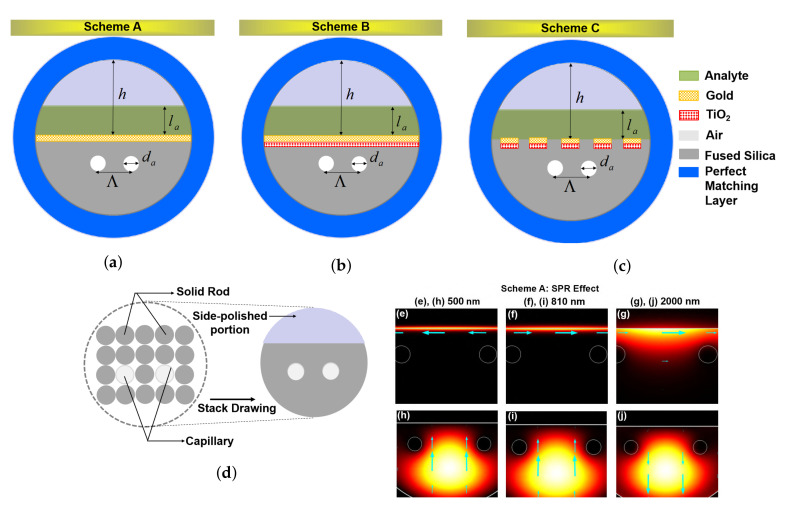
(**a**–**c**) schematic of the 2D cross section; (**d**) stacked preform of the proposed fiber; (**e**–**j**) the mode field distribution in the 2D cross-section of the proposed D-type PCF-RIBS for three different wavelengths, i.e., 500, 810, and 2000 nm, when $n_{a}$ = 1.4. The arrows indicate the direction of the electric field.

The proposed simplest structural D-type twin-hole PCF based plasmonic micro biosensors are characterized by using the finite element method (FEM). Figure 1a–c describe the geometrical cross-sections of the three different Schemes as A, B, and C (i.e., Au-coated, Au+TiO${}_{2}$-coated and Au+TiO${}_{2}$-grating assisted). In all the schemes, the diameter of the air-hole, $d_{a}$, is 2 μm and hole-to-hole spacing, Λ, is 5 μm. The thickness of the Au layer is analyzed from 40 to 60 nm in both schemes A and B. On the silica substrate, the air-holes are configured in order to trap the field to the center of the sensor and to strongly interact with Au. In Schemes B and C, TiO${}_{2}$ is used as a thin layer and as a grating, respectively, in-contact with a silica substrate for strong adherence. The essential material parameters such as RI of silica, Au, and TiO${}_{2}$ for the simulation are fetched from Refs. [21,22,23]. The layer thickness of Au and TiO${}_{2}$ in Schemes A and B are defined as $t_{g}$ and $t_{t}$. In Scheme C, Au and TiO${}_{2}$ grating thickness are defined as $t_{gG}$ and $t_{tG}$, respectively. The perfectly matched layer (PML), $t_{p}$, is a simulation boundary that absorbs the scattering waves from the fiber. An analyte layer thickness, $t_{a}$, 5 μm is considered. Here, $d_{a}$ and $t_{p}$ are followed similarly in Schemes A–C as shown in Figure 1a–c. Additionally, the crucial subset of fiber based SPR sensors consists of fibers with a discrete-metallic pattern, reinforcing LSPR, employed on the fiber D-shaped surface as shown in Figure 1c. LSPR excitation is permissive by the same restraints as propagating SPs that necessitate shallow interactions between the metal surface and analyte, allowing for the excitation of localized SPs at adjacent deep interactions. Consequently, LSPR based sensors may be demonstrated directly on the straight cleaved on the fiber D-shaped surface that enormously elucidates the sensor architecture. LSPR based sensors are an attractive platform owing to the relative simplicity of the practical realization. However, LSPR is known for being admissible sensitive to refractive index changes when compared with sensors based on propagating plasmons. The stacking capillary diagram of the proposed PCF-RIBS is depicted in Figure 1d. It can be fabricated using capillary stacking technique [24]. The D-shaped portion of the fiber can be realized by the side-polishing technique [25] and, in this work, the polishing height, *h*, is 12 μm. The CVD technique has been adopted for coating metal as thin film and grating in nano-scale [26]. A CVD may be employed for a wide variety of base materials such as ceramics, glass, metals, and metal alloys as thin-films, and also helps to coat precision surfaces like metal-grating including the seal areas and internal surfaces. In addition, the CVD technique can withstand exposure to low, moderate, and high-temperature variations. In particular, the CVD remains bonded in high-stress environments and when the surface flexes owing to high adhesion characteristics. Effective PML is simulated by applying the boundary condition for improving the accuracy of the results [27]. External D-type sensing is channelized on the corrugated surface of the proposed fiber so that core-guided field interacts closely with an analyte-sample from 1.33 to 1.45.

### 2.2. COMSOL 2D Modeling

The FEM by COMSOL MULTIPHYSICS has been employed to investigate the proposed sensing schemes in 2d geometry. The FEM is advantageous for waveguiding geometries [28], as it allows for characterizing the excited modes with an adequately modest computer memory requirements with a considerable mesh that allocates the particular structure of the sensor and their geometrical components. The design and computational analysis of the sensor schemes has been set to an extremely fine mesh size with the number of vertex elements of 24, number of boundary elements of 5231, number of elements of 118,680, and minimum element quality of 0.3203. In general, the FEM is utilized when it is requisite to vary the resolution so that the field is delineated over different regions of the simulation domain. Effective PML is simulated by applying the boundary condition for improving the accuracy of the results [28]. External D-type sensing is channelized on the corrugated surface of the proposed fiber so that core-guided field interacts closely with an analyte-sample from 1.33 to 1.45. As the proposed design has the most simple geometry, the practical realization may be achievable by the direct stack and draw method. With the reduced number of air holes, the proposed design is undoubtedly one of the simplest designs to be considered for fabricating an LSPR based PCF sensor to facilitate the rationality and feasibility of the proposed sensing schemes.

## 3. Investigation of Modes and Physical Mechanism

**Figure 2:**
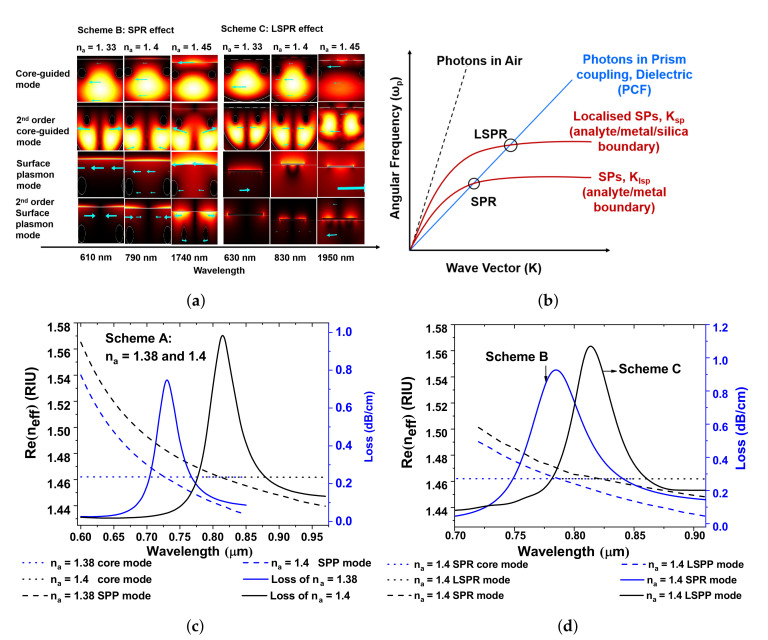
(**a**) mode field distribution in the cross-section of D-type twin-hole PCF-RIBS for the principle phase matching wavelength of $n_{a}$ = 1.33, 1.4, and 1.45. (**b**) dispersion relations of air (dashed line), fiber medium (blue), and three SPRs at different boundaries (red). The circle indicates the occurrence of SPR in three different circumstances; (**c**,**d**) are dispersion relation of the proposed PCF-RIBS for Scheme A and comparison of Schemes B and C (i.e., SPR and LSPR), respectively.

It is known that the coupling of plasmonic mode (PM) or the localized surface plasmon with the core-guided fundamental mode (FM) at a resonant wavelength is known as SPR in a PCF [5,18,19,20]. By characterizing the resonance condition in PCF-RIBS, the FM, PM, and SPR modes at three different wavelengths 500, 810, and 2000 nm are depicted in Figure 1e–j. For 500 nm, in the lower wavelength range (500 ≤λ≤ 2000 nm), the FM and PM are tightly confined when compared with that of the higher wavelength of 2000 nm. Figure 1 describes the 2D cross-sectional view of the proposed sensing schemes and indicates the polarization direction, showcasing an arrow plot. The arrow surface plots describe the wave vector directions for the incident field and the 2D cross-sectional mode wave vector of each order of modes. Maxwell’s equations framing up the normal component of the electric displacement field have to be continuous across interfaces between materials with different refractive indices. Henceforth, the normal component of the electric field needs to be higher in the material with the low refractive index. This may be employed to improve and confine the guided mode into a narrow domain with a low refractive index regarding the slot domain. The dimension of this low refractive index domain is on the orders of tens of nanometers which keeps the optical energy tightly confined to a narrow area giving a high optical energy density in the waveguide, as denoted in arrow plots. Figure 2a describes the mode field distribution of FM and PM for various analytes of $n_{a}$ = 1.33, 1.4 and 1.45. It is obvious that the field distribution of the FM and higher-order FM modes as well as PM and higher-order PM modes increases with increase in $n_{a}$, along with an increase in wavelength. This results in an increase in confinement loss of the modes. The FM and PM in Figure 2a demonstrate the phase matching conditions of Schemes B and C for $n_{a}$ = 1.33, 1.4, and 1.45, explaining the both SPR and localized SPR analysis. Figure 2b describes the two different SPR couplings that happen between different PM and the photons propagating in PCF. The SPR and LSPR mode couplings are clearly explained in Figure 2a. As the effective RI of the FM and SP is a function of resonant wavelength, the field distribution increases with an increase in $n_{a}$. Consequently, the ionization of electrons shows a large mode field distribution in LSPR, which improves the spectral sensitivity. Figure 2c depicts the propagation loss spectrum (solid curve), dispersion relation of the FM (dotted curve) and PM (dashed curve) of Scheme A i.e., the Au-coated D-type twin-hole PCF-RIBS for $n_{a}$ = 1.38 and 1.4. Here, the performances of the sensor such as confinement loss (CL), wavelength sensitivity (WS), and amplitude sensitivity (AS) are calculated using following the relations [5,18,19,20],

(1) $$\begin{array}{c} \alpha_{loss}=8.686\times \frac{2\pi }{\lambda }ℑ\left(n_{a}\right)\times 10^{4}, \end{array}$$

(2) $$S_{\lambda }\left[nm/RIU\right]=\frac{△\lambda_{peak}}{△n_{a}},$$

(3) $$S_{A}\left(\lambda \right)\left[RIU^{-1}\right]=-\frac{1}{\alpha (\lambda ,n_{a})}\frac{\partial \alpha (\lambda ,n_{a})}{\partial n_{a}},$$

where $ℑ(n_{a})$ is the imaginary part of the effective RI of the FM. $△\lambda_{peak}$ and $△n_{a}$ indicate the peak wavelength difference and change in analyte-RI, $\alpha (\lambda ,n_{a})$. $\partial \alpha (\lambda ,n_{a})$ is the overall loss, which is essentially the difference between two loss spectra. In Figure 2c, the resonance conditions occur at 730 nm (peak loss is 0.74749 dB/cm) and 810 nm (peak loss is 0.94107 dB/cm) for $n_{a}$ = 1.38 and 1.4. When $n_{a}$ is increased, the confinement loss increases with increase in wavelength and thus proves the capability of detecting the biological and chemical analytes. The reason for utilizing the TiO${}_{2}$-coating between Au and silica is to enhance the adhesion and easily flakes with light pressure [29]. To establish appropriate adhesion and to create a large number of electrons at the surface that generates a strong evanescent wave and attracts the fields from the core to interact strongly with the PM. This enhances the confinement loss, and it eventually improves the sensitivity. With this understanding, the Schemes B and C are followed as Au + TiO${}_{2}$-coated thin film for SPR analysis and Au + TiO${}_{2}$-grating assisted for LSPR investigation. A 30 nm coating between the silica and Au increases the confinement loss and broadens the loss peak. On the other hand, a grating of 30 nm provides a sharp loss peak which enhances the amplitude sensitivity. From Figure 2d, it is evident that the phase matching wavelength is 810 nm with its corresponding loss is 0.941 dB/cm for $n_{a}$ = 1.4. However, for a resonant wavelength of 780 nm, the corresponding loss is 1.129 dB/cm. This proves that the Scheme C i.e., LSPR based PCF-RIBS fetches the sharp loss peak and shifts the resonance condition to longer wavelength, which, in turn, increases the amplitude sensitivity.

## 4. Influence of Metals and Optimization

**Figure 3:**
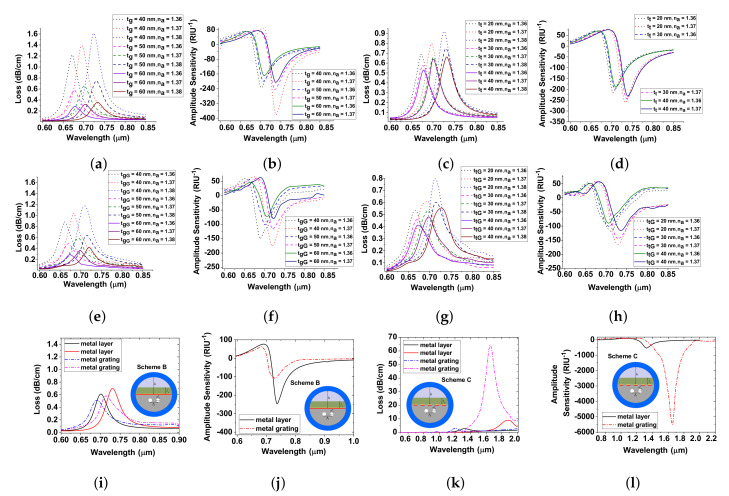
(**a**–**d**) confinement loss and amplitude sensitivity with $t_{g}$ and $t_{t}$ variations; (**e**–**h**) confinement loss and amplitude sensitivity with $t_{gG}$ and $t_{tG}$ variations; (**i**–**j**) confinement loss and amplitude sensitivity for SPR and LSPR at shorter wavelengths; (**k**–**l**) confinement loss and amplitude sensitivity for SPR and LSPR at longer wavelengths.

In order to understand the influence of various metals, we vary the thicknesses of Au and TiO${}_{2}$ by assigning the following physical parameters as $d_{a}$ = 2 μm, Λ = 5 μm and $t_{a}$ = 1200 nm. First, the Au layered thickness $t_{g}$ is varied as 40 nm, 50 nm, and 60 nm and the obtained characteristics are shown in Figure 3a,b. By considering the fabrication tolerance, we optimize the Au-layer thickness as 50 nm and the calculated wavelength sensitivity is 2000 nm/RIU and 3000 nm/RIU for $n_{a}$ of 1.36 and 1.37, respectively. For $t_{g}$ = 50 nm, the amplitude sensitivities are 199 RIU${}^{-1}$ and 242 RIU${}^{-1}$ for $n_{a}$ = 1.36 and 1.37, respectively. In this line, to improve the adhesion between the silica and Au, the TiO${}_{2}$ is introduced. The variation of loss spectra and amplitude sensitivity is illustrated in Figure 3c,d for various thicknesses of 20 nm, 30 nm, and 40 nm for $n_{a}$ = 1.36 and 1.37. It is obvious that TiO${}_{2}$ acts as a transition metal, and its RI is also high. Thus, Au + TiO${}_{2}$ coating increases the generation of large number of electrons at the surface, eventually creating a strong evanescent wave and attracts the mode field energy from the core to interact strongly with the surface plasmon mode. As a result, the spectral sensitivity is increased as the confinement loss is increased. The TiO${}_{2}$ coating with Au achieves a good adhesion and obviously exfoliates with the light pressure [29]. A thin TiO${}_{2}$ layer of 30 nm between Au and silica increases the confinement loss with enhanced FWHM as shown in Figure 3c. Nevertheless, the TiO${}_{2}$ layer restores the sharp loss peak, which is also responsible for enhancing the amplitude sensitivity as shown in Figure 3d. The calculated wavelength and amplitude sensitivities are presented in Table 1. It is evident that the LSPR provides larger amplitude sensitivity which is nearly 1.15 times over SPR where the plasmons are merely generated in a localized region from the entire surface, which also helps to achieve a sharper loss peak, as energy of the core-guided mode effectively interacts with the definite area of the surface.

In LSPR, the thickness of $t_{gG}$ and $t_{tG}$ of Au+TiO${}_{2}$-grating is optimized to be 50 nm and 30 nm, respectively. It is clear that the CL increases with an increase in $n_{a}$ and wavelength. Figure 3e,f depict the confinement loss and amplitude sensitivity when $t_{gG}$ is varied. Figure 3g,h describe the confinement loss and amplitude sensitivity when $t_{tG}$ is varied. At shorter wavelengths, the CL resonance conditions are larger for SPR based guidance over LSPR based guidance for $n_{a}$ = 1.37 and 1.38, as shown in Figure 3i,j. The AS shows deep peak sensing responses. At larger wavelengths (500 ≤λ≥2000 nm), the CL resonance conditions are larger for LSPR based guidance for $n_{a}$ = 1.4 and 1.45 as depicted in Figure 3k,l. Based on the above numerical results, we corroborate that the LSPR provides high sensing performances for the longer wavelengths. Furthermore, LSPR demands less cost-effectiveness for real-world applications.

## 5. Fabrication Tolerance

**Figure 4:**
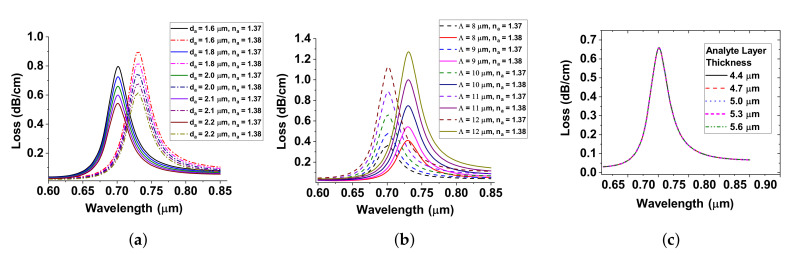
(**a**–**c**) confinement loss variation of diameter, pitch, and analyte layer as a function of wavelength of LSPR based twin-hole D-type PCF-RIBS.

Having optimized the thickness of $t_{gG}$ and $t_{tG}$ for LSPR, the next step is to optimize the diameter, pitch, and analyte layer. Here, we vary the air-hole diameter, $d_{a}$, from 1.6 μm to 2.4 μm and pitch, Λ, from 8 μm to 12 μm in steps of 0.2 μm when $n_{a}$ = 1.37 and 1.38. From Figure 4a,b, it is obvious that the confinement loss increases with increase in $n_{a}$ and also the resonant peak wavelength gets shifted to the larger wavelength. From Figure 4c, it is clear that the twin-hole D-type PCF-RIBS experiences low loss when $t_{a}$ is smaller and the loss peaks remain similar as variation is negligible when $t_{a}$ is increased.

## 6. Analysis of Sensitivity and Linearity

**Figure 5:**
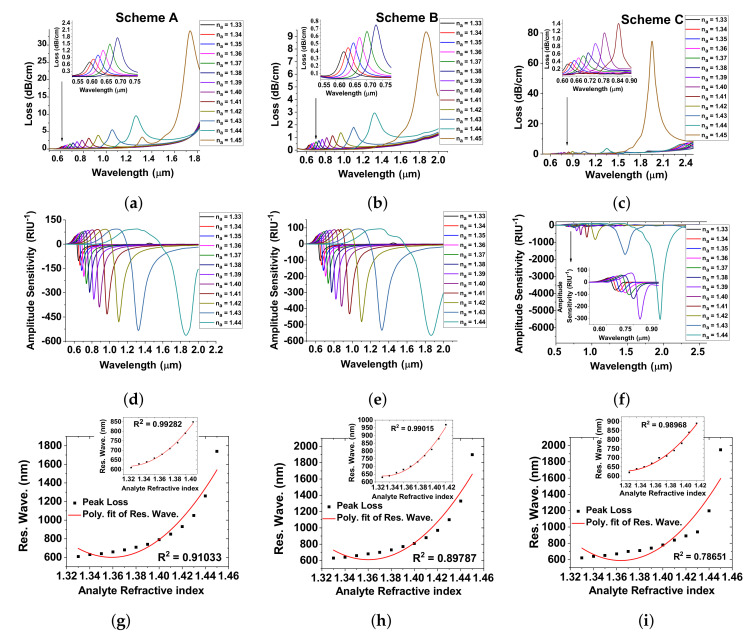
Confinement loss, amplitude sensitivity and fiber linearity having optimized geometrical parameters of the proposed D-type twin-hole PCF-RIBS as Scheme A: (**a**,**d**,**g**); Scheme B: (**b**,**e**,**h**); and Scheme C: (**c**,**f**,**i**).

The sensing performances such as confinement loss, amplitude sensitivity, and linearity of the proposed twin-hole D-type PCF-RIBSs are depicted in Figure 5a–i for various analytes, $n_{a}$ = 1.33 to 1.45.

It is obvious that the sensor is highly sensitive with the change of analyte-RI environment as it provides the negligible CL. As wavelength increases, the CL increases with increase in analyte-RI. From Figure 5a, we notice an extremely low CL from visible to near-IR resonance wavelengths for $n_{a}$ = 1.33 to 1.45. Scheme A achieves the WS of 48,000 nm/RIU for the analyte-RI environment of 1.44 to 1.45. Additionally, it is clear from Figure 5a that the confinement loss resonance peak broadens for an analyte-RI of 1.45 that leads to low amplitude sensitivity around 761 RIU${}^{-1}$ as shown in Figure 5d. When TiO${}_{2}$ is introduced to improve the adhesion between the Au and dielectric, it enriches the core-guided mode confinement as in Scheme B and improves the WS to 52,000 nm/RIU for the $n_{a}$ of 1.44 to 1.45 as shown in Figure 5b. In the presence of TiO${}_{2}$, the CL is low and it provides the AS of 560 RIU${}^{-1}$ as depicted in Figure 5e. It is obvious that the increase in confinement loss with increase in $n_{a}$ is owing to the lower penetration of the electromagnetic field towards the core-guided mode and higher penetration towards the localized SP modes. As a result, there is a resonance peak shift. Here, we achieve a maximum wavelength sensitivity of 75,000 nm/RIU as depicted in Figure 5c and high amplitude sensitivity of 5503 RIU${}^{-1}$ as explained in Figure 5f. Furthermore, we characterize the sensors for an analyte-RI from 1.33 to 1.45 as most of the biochemical and biomedical analytes fall in the RI range of 1.33–1.45 [15,16,17]. Hence, we characterize the sensor for an RI up to 1.45 as a further increase might result in incorrect detection of analytes due to such broadening of the loss peak.

In addition, the linearity of the proposed sensors of Schemes A, B, and C is depicted in Figure 5g–i, respectively. It is clear that the resonant wavelength increases with an increase in analyte-RI environment of 1.33 to 1.45. Here, the Scheme A achieves a good linearity performance of R${}^{2}$ = 0.91033 for the analyte-RI environment of 1.33 to 1.45 as shown in Figure 5g. In addition, the R${}^{2}$ value becomes 0.99282 for RI between 1.33 to 1.42, which is shown as the inset in Figure 5g. Figure 5h represents the sensor Scheme B whose R${}^{2}$ value is 0.89787 for $n_{a}$ = 1.33 to 1.45. However, the R${}^{2}$ value is 0.99015 for RI between 1.33 to 1.42. For Scheme C, the R${}^{2}$ value is 0.78651 for RI between 1.33 and 1.45. However, the R${}^{2}$ value turns 0.98968 for RI between 1.33 and 1.42 as described in Figure 5i. Therefore, the R${}^{2}$ value proves that the proposed sensing schemes are efficient to analyze the analyte-RI environment of 1.33 to 1.45 of chemical and biological samples.

In addition, we determine the sensing performances, such as R,SNR,δn and FOM, of the proposed sensors by using the following relations [30]:

(4) $$\begin{array}{c} R[RIU]=\Delta n_{a}\times \frac{\Delta \lambda_{min}}{\Delta \lambda_{peak}}, \end{array}$$

(5) $$\begin{array}{c} SNR=\frac{\Delta \lambda_{res}}{\Delta \lambda_{1/2}}, \end{array}$$

(6) $$\begin{array}{c} \delta n=\frac{\Delta \lambda_{1/2}}{1.5{\left(SNR\right)}^{0.25}}, \end{array}$$

(7) $$\begin{array}{c} FOM=\frac{S}{\Delta \lambda_{1/2}}, \end{array}$$

where $\Delta \lambda_{1/2}$ and $\Delta \lambda_{res}$ are the FWHM and loss peak wavelength difference. The sensing performances of the proposed sensors i.e., Schemes A, B, and C are tabulated in Table 1. Of these three schemes, the proposed sensors with low confinement loss achieve a high sensor length in the range of cm that is suitable for the experimental realization. In addition, the sensing performances of the proposed sensors schemes are compared with the existing sensors in Table 2. From the detailed analysis of comparison, we find that that the proposed sensors’ schemes hold the simplest geometry capable of detecting a large number of samples and also shows the enhanced efficiency of sensing performances.

## 7. Conclusions

In this work, we have numerically designed and investigated a low loss, highly sensitive three different schemes of metal influenced D-type twin-hole PCF-RIBSs for SERS response in the visible and mid-IR spectral regions. By using the finite element method, the simplest proposed D-type twin-hole PCF-RIBS has been demonstrated by LSPR and SPR response with optimized structural parameters for the biological and chemical detection. Hence, the proposed LSPR D-type PCF-RIBS has been able to achieve a maximum sensing performance compared to Schemes A and B for a wide range of an analyte-refractive index from 1.33 to 1.45 for and for a wide range of wavelengths from 500 to 2000 nm. Therefore, we are of the opinion that the D-type twin-hole PCF-RIBS as depicted in Figure 1d, with the inherent advantages that have been delineated, besides the ease of experimental fabrication by the well-known capillary stacking method, can be a good candidate for its enhanced sensitivity and precise detection of biological as well as chemical analytes. 

## Figures and Tables

**Table 1:** Sensing performance analysis of the proposed LSPR based D-type twin-hole PCF-RIBS.

Analyte-RI	Res. Wave. (nm)	Wave. Sensitivity (nm/RIU)	Resolution (RIU)	Amp. Sensitivity (RIU${}^{-1}$)	SNR	Detection Limit	FWHM (nm)	FOM
**Scheme A-SPR PCF-RIBS**								
1.33	610	2000	5 × 10${}^{-5}$	99	0.5	31	40	50
1.34	630	1000	1 × 10${}^{-4}$	134	0.25	36	39	25
1.35	640	2000	5 × 10${}^{-5}$	170	0.55	27	36	55
1.36	660	2000	5 × 10${}^{-5}$	223	0.55	27	36	55
1.37	680	3000	3.33 × 10${}^{-5}$	274	0.83	25	36	83
1.38	710	3000	3.33 × 10${}^{-5}$	353	0.83	25	37	81
1.39	740	5000	2 × 10${}^{-5}$	407	1.38	22	36	138
1.4	790	6000	1.66 × 10${}^{-5}$	493	1.39	26	43	139
1.41	850	8000	1.25 × 10${}^{-5}$	560	1.45	33	55	145
1.42	930	12000	8.33 × 10${}^{-6}$	644	1.90	35	63	190
1.43	1050	21000	4.76 × 10${}^{-6}$	720	2.44	45	86	244
1.44	1260	48000	2.08 × 10${}^{-6}$	761	3.72	61	129	372
1.45	1740	N/A	N/A	N/A	N/A	N/A	N/A	N/A
**Scheme B-SPR PCF-RIBS**								
1.33	630	1000	1 × 10${}^{-4}$	102	0.24	38	41	21
1.34	640	2000	5 × 10${}^{-5}$	124	0.54	28	37	54
1.35	660	2000	5 × 10${}^{-5}$	153	0.52	29	38	52
1.36	680	2000	5 × 10${}^{-5}$	199	0.52	29	38	52
1.37	700	3000	3.33 × 10${}^{-5}$	242	0.76	27	39	76
1.38	730	4000	2.5 × 10${}^{-5}$	280	0.95	28	42	95
1.39	770	4000	2.5 × 10${}^{-5}$	340	0.95	28	42	95
1.4	810	7000	1.42 × 10${}^{-5}$	380	1.32	32	53	132
1.41	880	8000	1.25 × 10${}^{-5}$	431	1.40	34	57	140
1.42	960	14000	7.14 × 10${}^{-6}$	476	1.75	46	80	175
1.43	1100	23000	4.34 × 10${}^{-6}$	529	2.09	60	110	209
1.44	1330	52000	1.9 × 10${}^{-6}$	560	3.04	86	171	304
1.45	1850	N/A	N/A	N/A	N/A	N/A	213	N/A
**Scheme C-LSPR PCF-RIBS**								
1.33	620	2000	5 × 10${}^{-5}$	69	0.43	16	46	43
1.34	640	1000	1 × 10${}^{-4}$	76	0.20	10	49	20
1.35	650	2000	5 × 10${}^{-5}$	82	0.43	16	46	43
1.36	670	3000	3.33 × 10${}^{-5}$	89	0.63	22	48	63
1.37	700	1000	1 × 10${}^{-4}$	107	0.21	10	48	21
1.38	710	3000	3.33 × 10${}^{-5}$	138	0.6	23	50	60
1.39	740	4000	2.5 × 10${}^{-5}$	330	0.83	27	48	83
1.4	780	6000	1.6 × 10${}^{-5}$	532	1.58	36	38	158
1.41	840	5000	2 × 10${}^{-5}$	702	1.47	30	34	147
1.42	890	5000	2 × 10${}^{-5}$	835	1.04	33	48	104
1.43	940	26000	3.84 × 10${}^{-6}$	1638	4.81	117	54	481
1.44	1200	75000	1.33 × 10${}^{-6}$	5503	9.03	288	83	904
1.45	1950	N/A	N/A	N/A	N/A	N/A	137	N/A

**Table 2:** Comparison of the proposed LSPR based D-type twin-hole PCF-RIBS’ performance with current sensors.

Ref.	Fiber Structure	RI-Range	Wave. Sensitivity (nm/RIU)	Resolution (Wave. Int.) (RIU)	Amp. Sensitivity (RIU${}^{-1}$)	Resolution (RIU)
[4]	SPR based D-shaped PCF	1.36–1.38	3340	5.98 × 10${}^{-6}$	693	2.84 × 10${}^{-5}$
[15]	SPR based D-shaped MOF with hollow core	1.33–1.34	2900	N/A	120	N/A
[16]	SPR based D-shaped PCF with laterally accessible hollow-core	1.46–1.47	7200	N/A	91	N/A
[17]	Three D-shaped holes SPR-PCF	1.33–1.39	10,100	9.9 × 10${}^{-6}$	N/A	N/A
[31]	Graphene-Au coated D-shaped optical fiber	1.33–1.39	4391	2.28× 10${}^{-5}$	1139	8.78× 10${}^{-6}$
[32]	D-shaped nanoscale silver strip SMF	1.38–1.42	3240	3.08× 10${}^{-5}$	192	N/A
[33]	Double-core D-type PCF	1.30–1.33	12,000	1.01 × 10${}^{-5}$	N/a	N/A
[34]	Quasi D-shaped nanoscale silver strip SMF	1.33–1.42	3877	2.58 × 10${}^{-5}$	1236	8.1 × 10${}^{-6}$
[35]	Gold grating assisted SPR-D-shaped SMF	1.34–1.38	7500	1.31 × 10${}^{-5}$	67,608	N/A
[36]	D-shaped SPR PCF	14,136–14,154	50,000	4 × 10${}^{-4}$	1266.67	N/A
[37]	Titanium nitride coated SPR D-shaped PCF	1.44–1.48	16,275	N/A	20,625	N/A
[38]	D-shaped SPR PCF	1.36–1.39	66,666.67	9.66 × 10${}^{-4}$	1488.82	N/A
**Proposed work**	Twin-hole D-type PCF-RIBS	1.33–1.45	75,000	1.33 × 10${}^{-6}$	5503	1.81 × 10${}^{-7}$

## Abbreviations

The following abbreviations are used in this manuscript:

SERS	Stimulated Emission of Raman Scattering
PCF	Photonic Crystal Fiber
RIBS	Refractive Index Based Sensor
Au	Gold
TiO${}_{2}$	Titanium-di-Oxide
SPR	Surface Plasmon Resonance
LSPR	Localized Surface Plasmon Resonance
SPM	Surface Plasmonic Mode
LSPM	Localized Surface Plasmon Mode
AUI	Analyte under Investigation
n${}_{a}$	Analyte-Refractive Index
LOD	Limit Of Detection
PM	Plasmonic Mode
FM	Fundamental Mode
CL	Confinement Loss
WS	Wavelength Sensitivity
AS	Amplitude Sensitivity
SNR	Signal to Noise Ratio
FWHM	Full-Width at Half Maximum
FOM	Figure of Merit
δn	Detection Limit
